# P-2084. HIV Viewpoints: Survey on the Treatment Experiences of People Living with HIV in Canada, Mexico, and the United States

**DOI:** 10.1093/ofid/ofaf695.2248

**Published:** 2026-01-11

**Authors:** Xavier Guillaume, Robin Barkins, Marcel Dams, Maureen Owino, Carlos Saucedo, Yun-Chung Lu, Amina Omri, Larkin Callaghan, Michael Bogart, Kesha O’Reilly, Megan Dunbar

**Affiliations:** Oracle Life Sciences, Paris, Ile-de-France, France; To Restore, Unite, Support, and Transform, Los Angeles, California; Aidshilfe NRW e.V., Cologne, Nordrhein-Westfalen, Germany; York University, Toronto, Ontario, Canada; Agenda LGBT A.C, Mexico City, Estado de México, Mexico; We As One Association, Taipei City, Taipei, Taiwan; Oracle Life Sciences, Paris, Ile-de-France, France; Gilead Sciences, Inc., Foster City, California; Gilead Sciences, Inc., Foster City, CA, United States, Foster City, California; Gilead Sciences, Inc., Foster City, California; Gilead Sciences, Forest City, CA

## Abstract

**Background:**

Understanding the diverse experiences of people with HIV (PWH) is crucial for enhancing engagement in care and improving long-term treatment outcomes.

**Figure:**
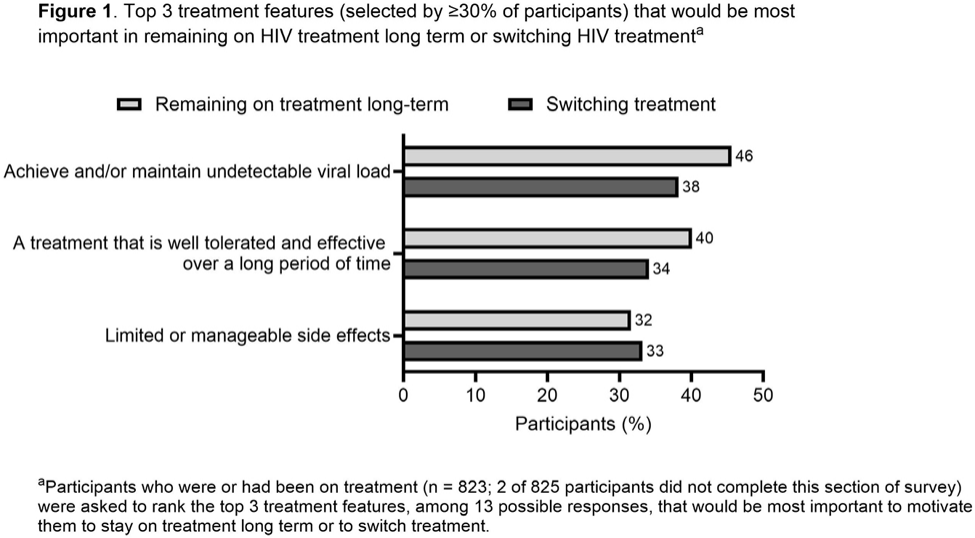

**Figure:**
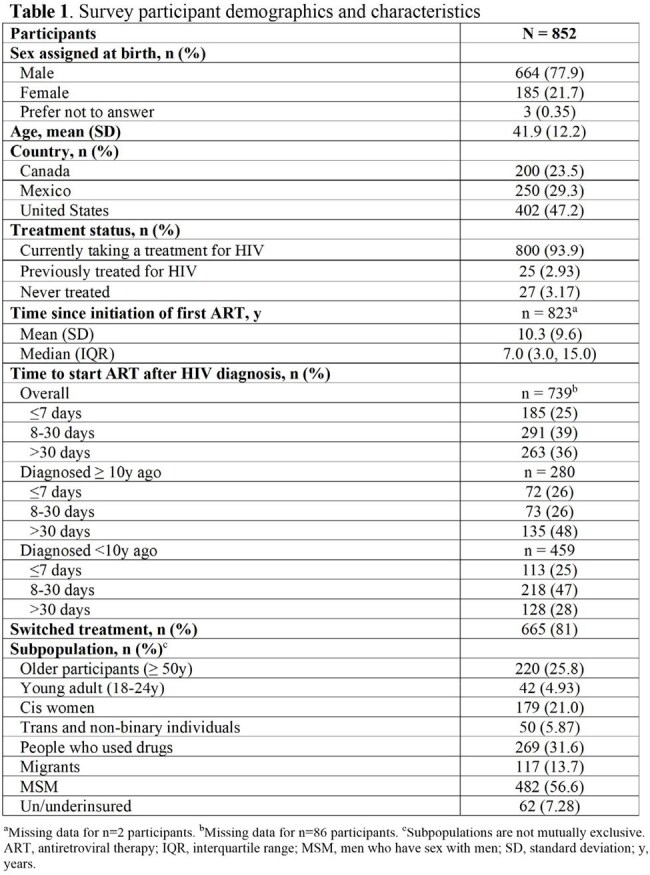

**Methods:**

A 45-minute, cross-sectional, online survey was co-developed by investigators and community advocates from Canada, Mexico, and the US and translated into local languages. The survey captured treatment experiences of PWH across the HIV care continuum. Participants ≥ 18y were recruited through patient databases, patient panels, advocacy groups, and physician referrals.

**Figure:**
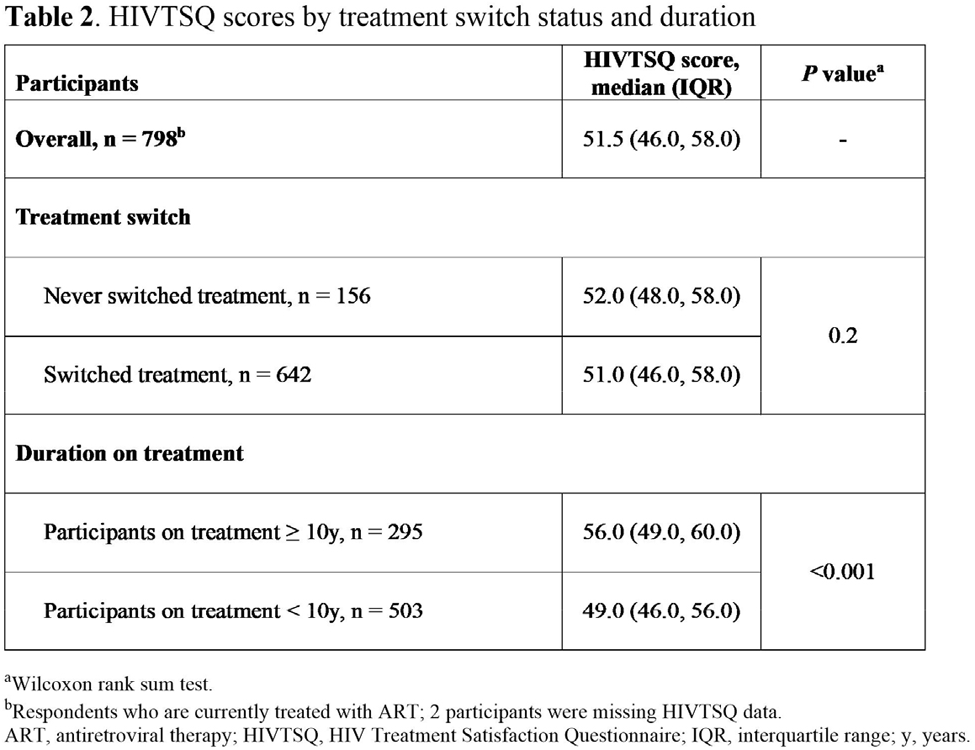

**Results:**

Among 852 participants, 24% were in Canada, 29% in Mexico, and 47% in the US (Table 1). Most participants (97%) were currently on or had previously taken antiretroviral therapy (ART), with the majority (77%) of those currently treated taking single-tablet oral daily ART. Among those who were or had been on ART, 36% initiated treatment >30 days after diagnosis (Table 1), including 48% of those diagnosed ≥ 10 years ago and 28% of those diagnosed < 10 years ago. Top reasons for delayed initiation were fear of potential side effects (29%), needing time to accept HIV diagnosis (28%), and physician recommendation based on CD4 count (25%).

Difficulties with treatment adherence were reported by 16% of participants taking oral ART and 13% taking injectable ART. The most important treatment features identified for staying on treatment long-term or for switching were that the treatment allowed PWH to achieve/maintain an undetectable viral load, was well tolerated and effective over a long period of time and had limited or manageable side effects (Figure 1). The median HIV Treatment Satisfaction Questionnaire status version score was 51.5/60.0 overall and was significantly higher for PWH had been on treatment for ≥ 10 years (56.0/60.0) compared with those who had been on treatment for < 10 years (49.0/60.0; Table 2).

**Conclusion:**

Participants reported high satisfaction with ART and identified treatment effectiveness, long-term safety, and side effects as top considerations for remaining on or switching HIV medication. A substantial proportion of participants delayed starting treatment. These factors highlight important considerations for supporting PWH to remain engaged in care and take medication as prescribed.

**Disclosures:**

Xavier Guillaume, n/a, Oracle Life Sciences, commissioned by Gilead Sciences, Inc.: Employee Amina Omri, n/a, Oracle Life Sciences, commissioned by Gilead Sciences, Inc.: Employee Larkin Callaghan, n/a, Gilead Sciences, Inc.: Employee|Gilead Sciences, Inc.: Stocks/Bonds (Public Company) Michael Bogart, n/a, Gilead Sciences, Inc.: Employee|Gilead Sciences, Inc.: Stocks/Bonds (Public Company) Kesha O'Reilly, n/a, Gilead Sciences, Inc.: Employee|Gilead Sciences, Inc.: Stocks/Bonds (Public Company) Megan Dunbar, PhD, Gilead Sciences, Inc.: Employee|Gilead Sciences, Inc.: Stocks/Bonds (Public Company)

